# Description of optochin-resistant *Streptococcus pneumoniae* due to an uncommon mutation in the* atpA* gene and comparison with previously identified *atpC* mutants from Brazil

**DOI:** 10.1038/s41598-021-87071-8

**Published:** 2021-04-12

**Authors:** Aline R. V. Souza, Sandrine E. C. M. de Pina, Natália S. Costa, Felipe P. G. Neves, Vânia L. C. Merquior, José Mauro Peralta, Tatiana C. A. Pinto, Lúcia M. Teixeira

**Affiliations:** 1 Instituto de Microbiologia Paulo de Goes, Universidade Federal Do Rio de Janeiro, Rio de Janeiro, RJ 21941-902 Brazil; 2 Instituto Biomédico, Universidade Federal Fluminense, Niterói, RJ 24210-130 Brazil; 3 Departamento de Microbiologia, Imunologia e Parasitologia, Universidade do Estado do Rio de Janeiro, Rio de Janeiro, RJ 20551-030 Brazil; 4 Present Address: Instituto de Biofísica Carlos Chagas Filho, Universidade Federal do Rio de Janeiro, Rio de Janeiro, RJ 21941-902 Brazil

**Keywords:** Microbiology, Molecular biology

## Abstract

Optochin susceptibility testing is a major assay used for presumptive identification of *Streptococcus pneumoniae*. Still, atypical optochin-resistant (Opt^r^) pneumococci have been reported and this phenotype has been attributed to nucleotide substitutions in the genes coding for the F_0_F_1_ATPase. While substitutions in the *atp*C gene (c-subunit of ATPase) are more common and better characterized, data on mutations in the *atp*A (a-subunit) are still limited. We have characterized five Opt^r^ isolates presenting alterations in the *atp*A (Trp**206**Cys in four isolates and Trp**206**Ser in one isolate), constituting the first report of such mutations in Brazil. Most of the Opt^r^ isolates consisted of heterogeneous populations. Except for Opt MICs and the nucleotide changes in the *atp*A gene, Opt^r^ and Opt^s^ subpopulations originating from the same culture had identical characteristics. In addition, we compared phenotypic and genetic characteristics of these *atp*A mutants with those of *atp*C mutants previously identified in Brazil. No structural alterations were detected among predicted proteins, regardless of mutations in the coding gene, suggesting that, despite the occurrence of mutations, protein structures tend to be highly conserved, ensuring their functionalities. Phylogenetic analysis revealed that atypical Opt^r^ strains are true pneumococci and Opt resistance does not represent any apparent selective advantage for clinical isolates.

## Introduction

*Streptococcus pneumoniae* is a leading cause of invasive diseases among children and the elderly associated with considerable mortality and economic burden^1^. Although infant mortality rates have been declining since 2000 due to broader access to pneumococcal vaccines, pneumococcal pneumonia remains as a major cause of child mortality^2^. Thus, pneumococcal infections require fast and accurate diagnosis. Conventional laboratory identification of *S. pneumoniae* usually relies on one major characteristic of this species: the susceptibility to optochin (Opt). Opt susceptibility testing can distinguish the human pathogen *S. pneumoniae* from other alpha-hemolytic species, such as *Streptococcus mitis* and *Streptococcus pseudopneumoniae*^3^.

Nevertheless, isolation of Opt-resistant (Opt^r^) pneumococcal has been reported in different geographical areas^4–11^, drawing attention to the potential for misidentification of this relevant pathogen. Point mutations in the *atp*C gene are usually attributed to this atypical phenotype; the *atp*C gene codes for the c-subunit of the bacterial F_0_F_1_ATPase*,* the molecular target of optochin^4–6,8,9,11–13^. Alterations in other subunits of this molecule are rare, and only two reports on Opt^r^ clinical strains of *S. pneumoniae* presenting mutations in the *atp*A gene (coding for the a-subunit of the bacterial F_0_F_1_ATPase) are available to date^5,13^.

Our group has reported the occurrence of previously recognized as well as novel mutations in the *atp*C gene among a set of 26 Opt^r^ pneumococcal isolates in Brazil^6,9^. In the present study, we describe five additional Opt^r^ isolates presenting alterations in the *atp*A gene, constituting the first report of such unusual mutations in pneumococcal isolates from our country. In addition, we extended the spectrum of methodologies used and compared phenotypic and genetic characteristics of all Opt^r^ pneumococcal strains described in Brazil up to now, including both *atp*C and *atp*A mutants.

## Results

### Strain identification and characterization of Opt resistance phenotypes

All five isolates described in the study had the following characteristics: they were gram-positive catalase-negative cocci; presented alpha-hemolysis on sheep blood agar plates; generated positive results in the bile solubility and in the latex agglutination tests; harbored the *lytA*, *ply* and *psaA* genes; and were resistant to Opt.

**Figure 1:**
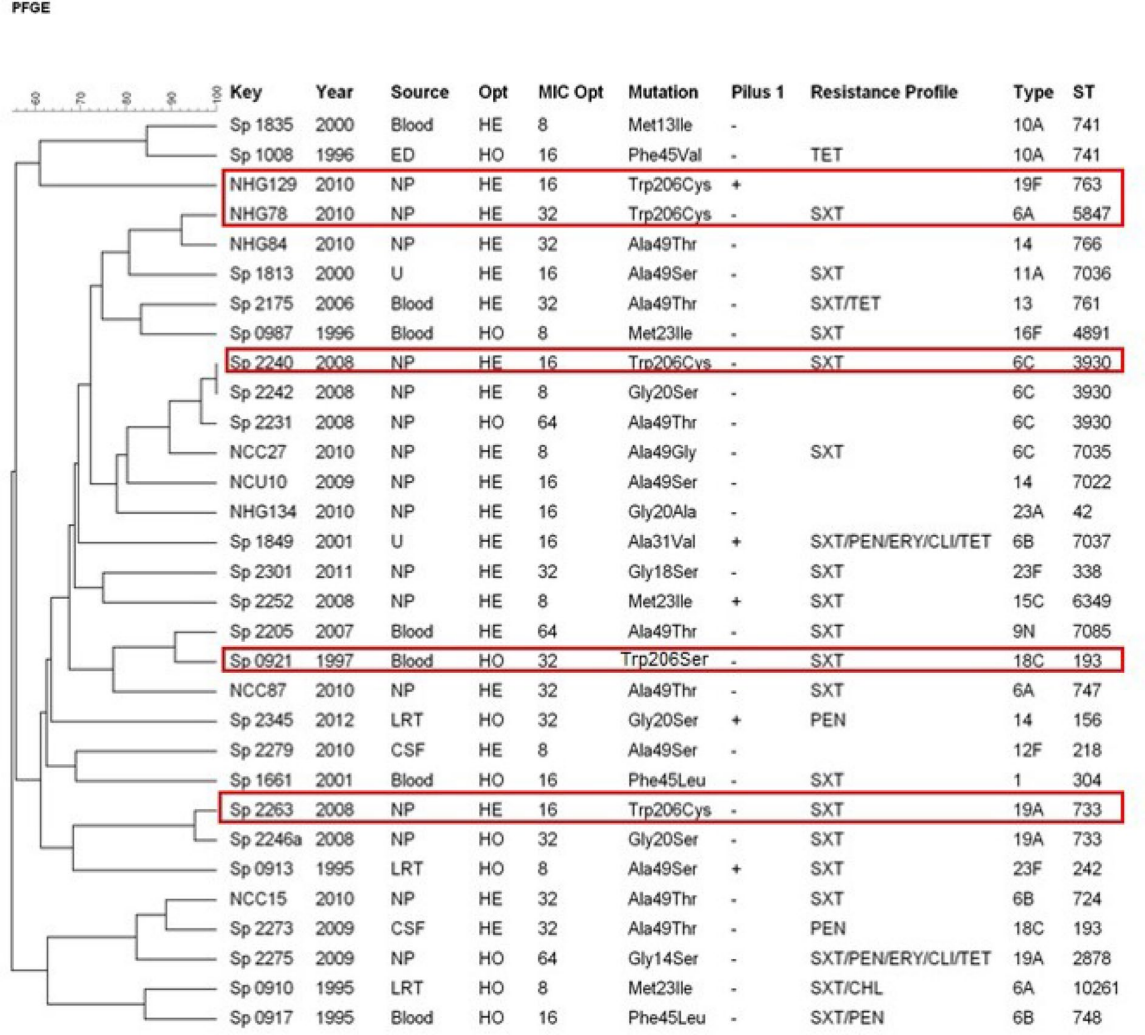
UPGMA dendrogram generated by using BioNumerics v.7.6 based on PFGE profiles of 31 optochin-resistant *Streptococcus pneumoniae* strains isolated in Brazil. The five a*tp*A gene mutants described in this work are marked in red. *Ala* alanine, *CLI* clindamycin, *CHL* chloramphenicol, *CSF* cerebrospinal fluid, *Cys* cysteine, *ED* eye discharge, *ERY* erythromycin, *Gly* glycine, *HE* heterogeneous, *HO* homogeneous, *Ile* isoleucine, *Leu* leucine, *LRT* low respiratory tract, *Met* methionine, *MIC* minimum inhibitory concentration, *NP* nasopharyngeal carriage, *Opt* optochin, *PEN* penicillin, *Phe* phenylalanine, *Ser* serine, *ST* sequence type, *SXT* sulfamethoxazole-trimethoprim, *TET* tetracycline, *Thr* threonine, *Trp* tryptophan, *Val* valine, *U* unknown.

Opt susceptibility testing, under both CO_2_-enriched or conventional atmospheres, revealed the occurrence of two phenotypes among the five Opt^r^ isolates. The first was represented by one isolate displaying no inhibition zone, consisting of a homogeneous Opt^r^ population. The second phenotype was represented by the other four isolates, showing inhibition zones > 14 mm but with colonies within, consisting of heterogeneous populations comprised by Opt^r^ and Opt-susceptible (Opt^s^) subpopulations. Opt MICs ranged from 16 to 32 μg ml among the five Opt^r^ strains evaluated (Fig. 1). The single Opt ^r^ homogeneous population had an Opt MIC of 32 μg/ml. Opt^r^ subpopulations derived from heterogeneous populations exhibited MICs between 16 and 32 μg/ml (Fig. 1); while Opt^s^ counterparts presented Opt MICs of 1 μg/ml. Opt MICs of Opt^s^ subpopulations were identical to those detected among optochin-susceptible reference strains of *S. pneumoniae* ATCC BAA-255 and ATCC 49619 (1 μg/ml). Opt MICs of reference strains of *S. pseudopneumoniae* ATCC BAA-960 and *S. mitis* ATCC 49456 were 8 μg/ml and 256 μg/ml, respectively.

In addition to the five Opt^r^ strains included in the present study, the 26 Opt^r^ isolates previously reported in Brazil^6,9^ were also subjected to identification by MALDI-TOF MS. All 31 strains were identified as *S. pneumoniae,* with scores between 2.089 and 2.452. Of note, Opt^r^ and Opt^s^ subpopulations derived from heterogeneous populations were also analyzed separately, and in all cases both subpopulations were identified as pneumococcus with scores higher than 2.0. However, reference strains *S. mitis* ATCC 49456 and *S. pseudopneumoniae* ATCC BAA-960 were also identified as *S. pneumoniae* by MALDI-TOF MS, with scores of 1.938 and 2.213, respectively.

### Sequencing of atpA gene

**Figure 2:**
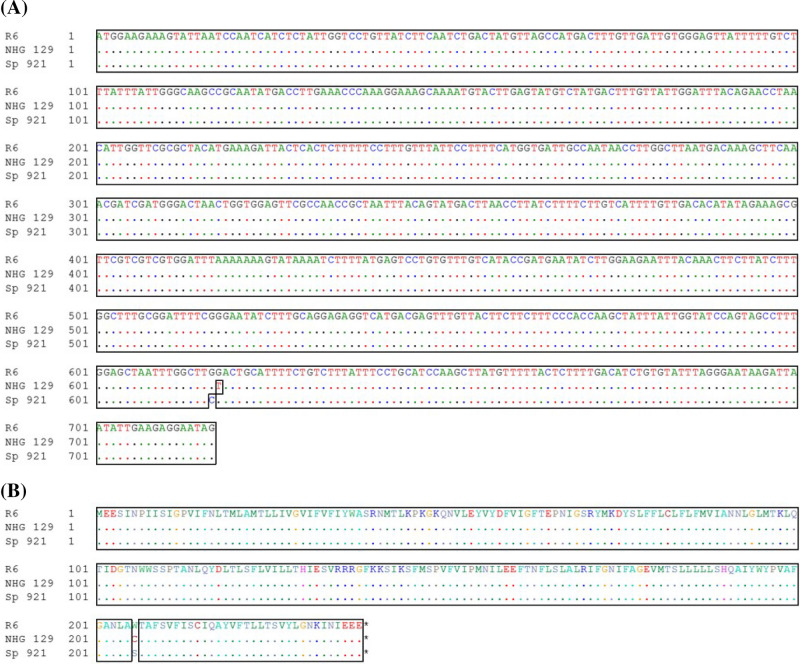
Alignment of representative *atp*A gene sequences observed among optochin-resistant (Opt^r^) *Streptococcus pneumoniae* isolates. (**A**) Nucleotide sequences of the *atp*A gene representing the two different base-substitutions observed among the five Opt^r^ isolates included in the present study. (**B**) Deduced amino acid sequences of the a-subunit of ATPase representing, respectively, the two different base-substitutions mentioned above. Optochin-susceptible reference strain *S. pneumoniae* R6 was included for comparative purposes. Alterations are outlined with black boxes.

Sequencing of *atp*A genes revealed single-base substitutions leading to amino acid modifications in codon 206 in all five strains, comprising four isolates with a Trp**206**Cys substitution and one isolate with a Trp**206**Ser substitution (Figs. 1 and 2A,B). Interestingly, the single strain with a Trp**206**Ser substitution was also the single strain homogenously resistant to optochin. All the other four showing the Trp**206**Cys modification consisted on heterogeneous populations. Of note, while Opt^r^ subpopulations derived from heterogeneous populations presented this alteration in codon 206, Opt^s^ counterparts had *atp*A gene sequences identical to those of optochin-susceptible reference strains of *S. pneumoniae* (ATCC BAA-255 and ATCC 49619).

### In silico prediction of c- and a-subunit 3D models

**Figure 3:**
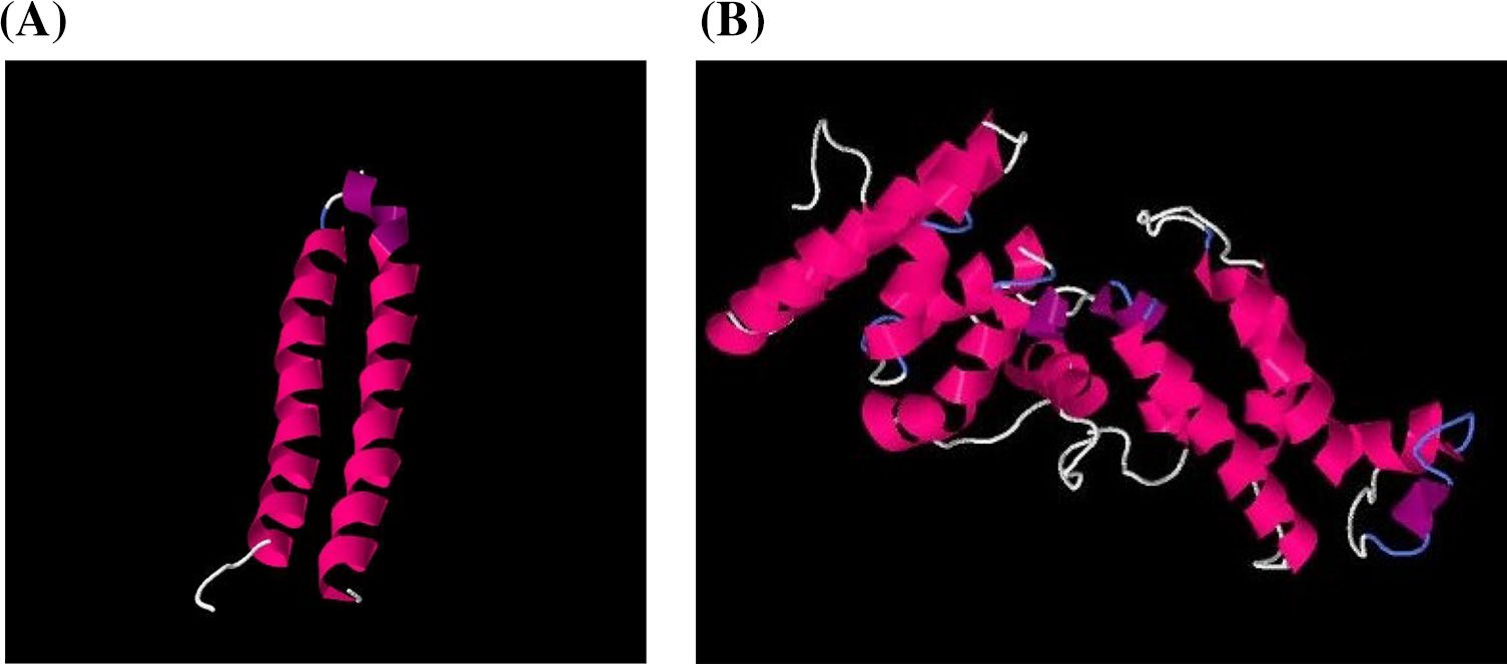
In silico predicted 3D models for c- (**A**) and a-subunits (**B**) of F0F1ATPase of *Streptococcus pneumoniae* created with Bioserf v.2.0. Alpha helices are colored in pink. All 31 optochin-resistant *S. pneumoniae* strains analyzed and the reference strains ATCC BAA-255 and ATCC 49619 generated identical models for both subunits.

The 3D structure of the a-subunit of F_0_F_1_ATPase predicted in silico from the *atp*A nucleotide sequences of the five Opt^r^ *S. pneumoniae* strains included in the study were identical to the models generated from Opt^s^ reference strains (*S. pneumoniae* ATCC BAA-255 and ATCC 49619), consisting of five transmembrane α-helices (Fig. 3A). Likewise, 3D models of the c-subunit, predicted from the *atp*C gene sequences of 26 Opt^r^ strains previously reported^6,9^ and retrieved from GenBank database, were indistinguishable from the ones of Opt^s^ reference strains (*S. pneumoniae* ATCC BAA-255 and ATCC 49619), consisting of two transmembrane α-helices (Fig. 3B). The structures were also identical between Opt^r^ and Opt^s^ subpopulations derived from heterogeneous populations.

### Phenotypic and molecular characterization of Opt^r^ strains

Opt^r^ and Opt^s^ subpopulations derived from heterogeneous populations showed identical results regarding capsular type, antimicrobial susceptibility profile, ST, PFGE, MLVA and PI-1 genes profiles.

The five Opt^r^ pneumococcal isolates included in this study belonged to serotypes 6A, 6C, 18C, 19A and 19F. All strains were susceptible to antimicrobial agents tested, except for sulfamethoxazole/trimethoprim (Fig. 1).

**Figure 4:**
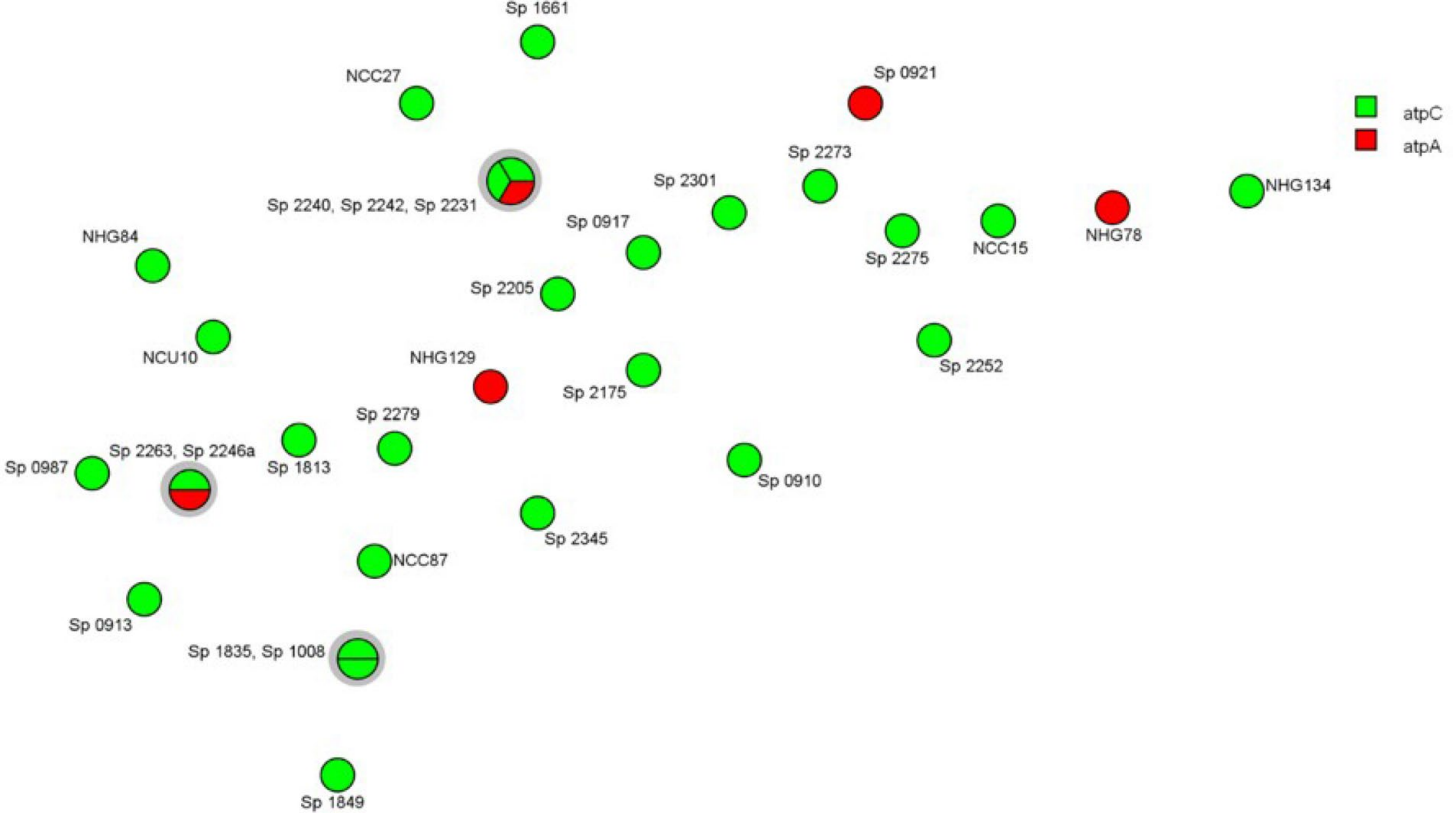
Minimum Spanning Tree generated by using BioNumerics v.7.6 and showing the genetic relationship among 31 optochin-resistant *Streptococcus pneumoniae* strains isolated in Brazil, according to the MLVA profiles. Each node represents one MLVA profile, and nodes are divided proportionaly to the number of isolates included in each MLVA profile. Clonal complexes (strains sharing five or more identical alleles) are shaded in grey. Absence of lines between nodes indicate they show three or more different alleles and are, therefore, genetically unrelated. Strains were differentiated by color according to the location of mutation (*atp*C or *atp*A; see legend).

**Figure 5:**
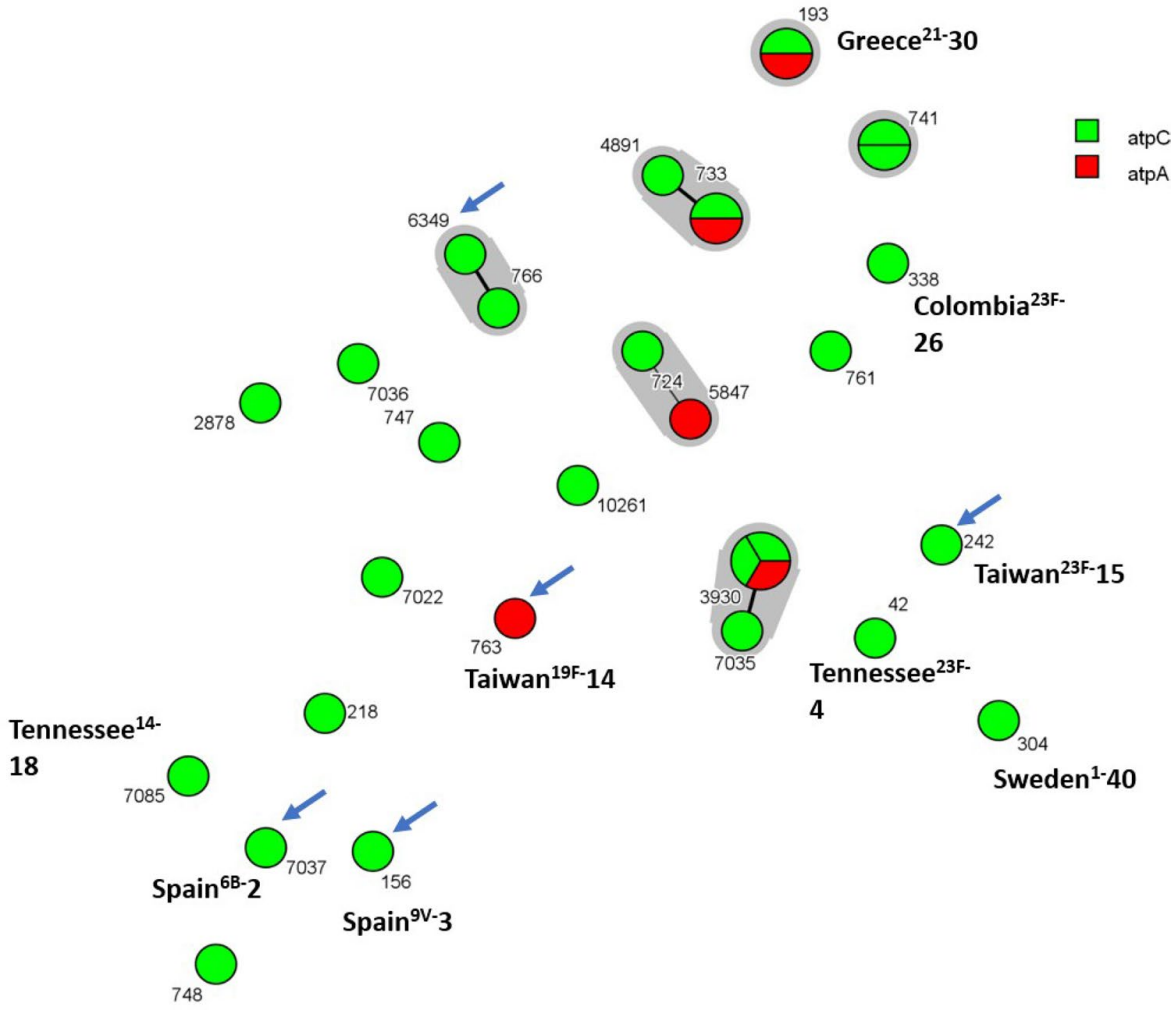
Minimum Spanning Tree generated by using BioNumericsv.7.6 and displaying the genetic relatedness inferred by MLST among 31 optochin-resistant *Streptococcus pneumoniae* strains isolated in Brazil. Each node represents one sequence type (ST), and nodes are divided proportionaly to the number of isolates included in the same ST. Internationally disseminated clones recognized by the PMEN and related to strains analyzed in the study are indicated. Clonal complexes (strains sharing five or more identical alleles) are shaded in grey. Absence of lines between nodes indicate they show three or more different alleles and are, therefore, genetically unrelated. Strains were differentiated by color according to the location of mutation (*atp*C or *atp*A; see legend). Strains harboring pilus type 1 genes are highlighted with a blue arrow.

Within the five *atp*A mutants, different PFGE patterns (Fig. 1), STs (193,733, 763, 3930 and 5847) (Figs. 1 and 5) and MLVA profiles (Fig. 4) were detected. When compared to the 26 Opt^r^ pneumococcal isolates previously described in Brazil, only three STs and two MLVA profiles were shared by two or more strains (Figs. 1, 4 and 5). It is also noteworthy that ten (32.25%) of all 31 Opt^r^ pneumococcal isolates analyzed belonged to internationally disseminated clones recognized by the PMEN (Fig. 5). Of note, there was no correlation between the type of *atp*C or *atp*A mutation with serotype, PFGE pattern, ST or MLVA type (Figs. 1, 4 and 5).

PI-1 genes were found in five (16.1%) of all 31 Opt^r^ isolates analyzed; of those, one had a mutation in the *atp*A gene while four had mutations in the *atp*C gene (Fig. 1). In addition, most of the PI-1 positive strains were related to PMEN clones.

### Phylogenetic analysis

**Figure 6:**
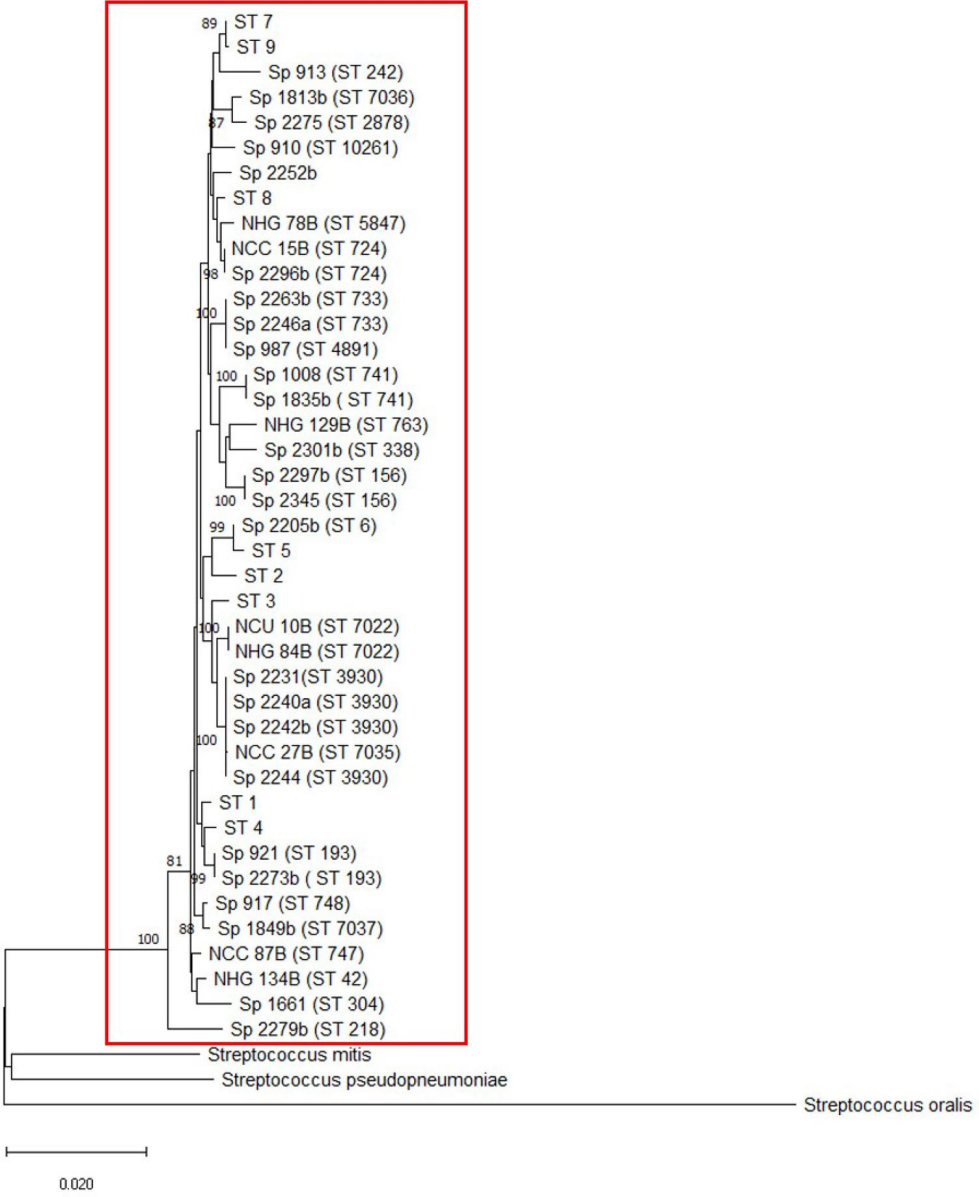
MLSA tree generated from the concatenated sequences of six housekeeping genes (*aro*E, *gdh*, *gki*, *rec*P, *spi* and *xpt*) showing the phylogenetic relationship among optochin-resistant *Streptococcus pneumoniae* (31 isolates), typical *S. pneumoniae* (8 isolates), *Streptococcus pseudopneumoniae* (strain 9230), *Streptococcus mitis* strain (SS691) and *Streptococcus oralis* (strain SS527). Scale bar indicates the nucleotide subtitution per site. Highlighted with a red box is the cluster comprising all 39 *S. pneumoniae* sequences analyzed, including the typical and the optochin-resistant strains.

Phylogenetic analysis revealed that all 31 Opt^r^ pneumococcal strains, including both *atp*A and *atp*C mutants, were clustered within a monophyletic group, with 100% of probability in an analysis using a bootstrap of 1000 (Fig. 6). This analysis also included typical *S. pneumoniae*, representative of Opt^s^ strains*.* On the other hand, *S. mitis*, *S. pseudopneumoniae* and *S. oralis* were clustered in different and more distantly related groups.

## Discussion

During the last decades, the occurrence of atypical optochin-resistant pneumococcal strains has been sporadically reported^4–10,12–14^, reinforcing the need of constant evaluation of traditional identification schemes for this relevant pathogen.

Different studies have described the occurrence of two phenotypes within collections of Opt^r^ *S. pneumoniae* isolates: a homogeneous type in which the isolate is fully resistant to optochin, and a heterogeneous type comprised by Opt^r^ and Opt^s^ subpopulations^5–7,9,12,13^. The distribution of each phenotype, however, seems to vary according to the geographical region. In Brazil, as well as in the United States^5^, the heterogeneous phenotype has been more commonly reported. Among the 26 Opt^r^ isolates previously reported from Brazil^6,9^, 16 (61.5%) were composed by heterogeneous populations (Fig. 1). On the other hand, in Portugal, homogenous Opt^r^ populations have been more frequently observed^7^. Of note, the only difference found between subpopulations derived from heterogeneous Opt^r^ populations, in this and previous studies, is the mutation in *atp*A or *atp*C gene. These observations suggest that an originally Opt^s^ population might at some point undergo a point mutation resulting in two different types of cells that, then, compose a heterogeneous Opt^r^ population. However, it remains unknown why in some strains these subpopulations tend to coexist while in others a homogeneous Opt^r^ phenotype occurs.

MALDI-TOF MS has gained large interest in the clinical microbiology setting and it has already been introduced in the routine of several clinical laboratories worldwide^15,16^. This methodology has been widely used for identification of a number of bacterial species, but the lack of discrimination among streptococcal species belonging to the mitis group is well documented^15,17^. Although alternative MALDI-TOF MS-based protocols for accurate differentiation of mitis group streptococci have been proposed, the majority depend on the analysis of mass spectra by trained staff, being unfeasible for application in many clinical laboratories, which usually rely on results generated by automated processing of spectra by dedicated softwares^16,18^. Therefore, when using MALDI-TOF MS for identification of *S. pneumoniae* in the clinical microbiology laboratory, it is mandatory to perform a complementary test, such as bile solubility or optochin susceptibility testing^19^. In this regard, laboratory personnel should be aware of the possible occurrence of atypical optochin-resistant pneumococcus, and preferentially perform an additional test if the isolate shows resistance to optochin.

Opt MICs found in this study, ranging between 16 and 32 μg/ml, were in agreement with MICs (8–64 μg/ml) observed among the 26 Opt^r^ Brazilian isolates previously described^6,9^ (Fig. 1), and also with those obtained for isolates from other locations^5,7^. Comparison of Opt^r^ and Opt^s^ MICs in heterogeneous populations revealed similar results to earlier studies, in which MICs of Opt^r^ subpopulations were 4–64 fold higher than those of Opt^s^ counterparts^5,9^. Moreover, no correlation between Opt MIC levels and Opt^r^ phenotype was detected since similar MIC levels were observed in both homogenous and heterogeneous populations. Likewise, no correlation between Opt MIC levels and type of mutation was observed, as previously reported^5,9,10^.

To the best of our knowledge, this is the first study to describe *atp*A mutants among clinical pneumococcal isolates in Brazil and also represents the largest and more extensively characterized collection of Opt^r^ *S. pneumoniae* clinical isolates reported in the literature. Earlier, only two Opt^r^ *S. pneumoniae* clinical isolates presenting mutations in *atp*A gene had been described^5,13^. The first was recovered from an individual’s nasopharynx in the United States, presented a heterogeneous phenotype, a Trp**206**Ser substitution, an Opt MIC of 32 μg/ml, and belonged to serotype 6B^5^. The second was recovered from a patient’s blood in Argentina, showed a homogeneous phenotype, a Trp**206**Cys modification, an Opt MIC of 64 μg/ml, and belonged to serotype 5^13^.

Our results reinforce the observation that mutations in the *atp*A gene are not correlated with specific clinical source or serotype, but also highlight that substitutions in this gene, regardless of any other characteristic, are always in the same position, the codon 206. This is quite different from what has been observed within *atp*C mutants, among which substitutions have been identified in 12 different codons of the c-subunit^4–6,8–10,13,14^. Still, despite the higher variability, around 50% of the 48 *atp*C mutants described to date show alterations in the same location, the codon 49, which has been suggested as a hot-spot for this type of mutation. Codon 206 in the a-subunit could act as a hot-spot as well, but this should be established as more *atp*A mutants are identified.

In silico predicted 3D models of a- and c-subunits indicate that, despite the occurrence of alterations at the primary level, 3D models of c- and a-subunits tend to be highly conserved, regardless of optochin susceptibility profile, which could probably confer the maintenance of enzyme functional activity and, consequently, the survival of bacteria. Although no structural alterations on predicted proteins were detected in the present study, it has been previously observed that mutations in the c-subunit led to inability of these bacteria to survive in acidic conditions, including survival inside of macrophages^20^. Mutations in the a-subunit of F0F1 ATPase, on the other hand, presented no implications regarding acid tolerance^20^. Nevertheless, additional studies are needed to clarify the impact of *atp*A and *atp*C mutations on pneumococcal fitness.

The variability of PFGE profiles, STs and MLVA types detected in the study indicate that optochin resistance is not a clonal-specific characteristic, in agreement with previous findings^7,9^. However, PMEN clones previously associated with the presence of PI-1^21^ and known to be circulating in Brazil in recent years^9,22–24^ were found among Opt^r^ strains, suggesting that highly successful clones can exhibit the atypical Opt^r^ phenotype, increasing the concern on possible consequences of the misidentification of these variants*.*

Phylogenetic analysis clearly shows that atypical Opt^r^ *S. pneumoniae* and typical pneumococci have evolved from the same ancestral (Fig. 6), while evolution that resulted in different streptococcal species occurred by an independent route. These data, in conjunction with the other results of this study, strongly suggest that Opt^r^ isolates are authentic *S. pneumoniae* that have diverged from typical strains by point mutations in the the *atp*C or *atp*A genes.

In conclusion, our results suggest that optochin resistance in *S. pneumoniae* is not related to a specific phenotypic or genetic characteristic, but is rather due to the random occurrence of mutations in the *atp*A or *atp*C genes. Even though mutations in a- or c-subunits of F_0_F_1_ ATPase can result in amino acid changes, their predicted 3D structures remain unaltered, ensuring survival of these strains. Phylogenetic analysis revealed that atypical Opt^r^ strains are true pneumococci and Opt resistance does not represent any apparent selective advantage for clinical strains.

## Methods

### Bacterial strains and identification tests

Five Opt^r^ *S. pneumoniae* isolates (Sp 921, Sp 2240, Sp 2263, NHG78 and NHG129) recovered from individuals living in Brazil were included in the present study, based on the finding that they showed no alterations in the *atp*C gene during initial screening tests, carried out as previously described^9^. Four isolates were obtained during nasopharyngeal carriage surveillance studies performed as approved by the ethics committees of the institutions involved. One isolate (Sp 921) was recovered from a blood culture taken as part of the standard patient care procedures, so a specific ethical approval was not needed. The isolates were subjected to conventional phenotypic identification tests, including observation of colony morphology and type of hemolytic activity on sheep blood agar plates; cellular characteristics as observed after Gram stain; Opt susceptibility, bile-solubility, and latex agglutination tests^3^. Identification was also performed by MALDI-TOF MS, using a Microflex LT equipment (Bruker Daltonics, Bremen, Germany), according to the manufacturer recommendations. For comparative purposes, a collection of 26 Opt^r^ pneumococcal isolates presenting mutations in the *atp*C gene, previously identified by our group^6,9^, was also included for further characterization by MALDI-TOF, MLSA, MLST, detection of PI-1 genes and in silico prediction of c-subunit 3D model. Reference strains of *S. pneumoniae* (ATCC BAA-255 and ATCC 49619), *S. pseudopneumoniae* (ATCC BAA-960) and *S. mitis* (ATCC49456) were included in all identification tests.

### Optochin susceptibility testing

Opt susceptibility was determined by disk diffusion testing according to standard procedures^3^. Optochin disks (BBL Taxo P Discs, BD, Sparks, MD, USA) were applied to the surface of 5% sheep blood agar plates (Plast Labor, Rio de Janeiro, RJ, Brazil) streaked with the isolates being tested. After overnight incubation at 36 °C, under both 5% CO_2_ and conventional atmospheres^25^, growth inhibition zones around the disks were measured. Isolates displaying inhibition zones ≥ 14 mm in diameter were identified as susceptible, while strains were considered resistant when showing zones < 14 mm or zones ≥ 14 mm but with colonies within. This last case was classified as heterogeneous population, composed by both Opt^r^ and Opt-susceptible (Opt^s^) subpopulations; all subsequent experiments were carried out separately for both subpopulations after obtaining pure cultures of each one. Additionally, optochin (Sigma Chemical Co., St. Louis, MO, USA) minimal inhibitory concentrations (MICs) were also determined, by the agar dilution method as previously described^9^.

### Sequencing of *atp*A gene

DNAs for all PCR reactions were obtained by using the Chelex 100 resin (Bio-Rad, Hercules, CA, USA) as described earlier^9^, followed by amplification of the *atp*A gene^11^. Products were purified with ExoSAP-IT (USB Affymetrix, Cleveland, OH, USA) and sequenced using ABI 3130 Genetic Analyzer (Applied Biosystems). Sequences were aligned and analyzed with Bioedit software v7.0.9.0^26^, using *atp*A nucleotide sequences of Opt^s^ reference strains (*S. pneumoniae* ATCC BAA-255 and ATCC 49619) as standards.

### In silico prediction of c- and a-subunit 3D models

Using the amino acid sequences translated from the nucleotide sequences obtained in this study, predicted 3D models of the a-subunit of F_0_F_1_ ATPase were designed by using the online tool Bioserf v2.0, available at PSIPRED Protein Sequence Analysis Workbench^27,28^. Predicted 3D models of the c-subunit were generated using the *atp*C gene sequences of 26 Opt^r^ strains previously reported^6,9^ retrieved from the GenBank database (accession numbers KC513927 to KC513948); *atp*C and *atp*A nucleotide sequences of Opt^s^ reference strains (*S. pneumoniae* ATCC BAA-255 and ATCC 49619) were included for comparative purposes.

### Determination of capsular types

Capsular types were determined by either PCR^29^ or the standard Quellung reaction^30^.

### Antimicrobial susceptibility testing

Susceptibility to antimicrobial agents was evaluated by the agar diffusion test according to CLSI recommendations and interpretative criteria^31^. The following agents were tested: chloramphenicol, clindamycin, erythromycin, levofloxacin, oxacillin, rifampicin, tetracycline, sulfamethoxazole-trimethoprim and vancomycin (all from Oxoid, Basingstoke, Hampshire, United Kingdom). *S. pneumoniae* ATCC 49619 was used for quality control.

### Detection of virulence-associated genes

The presence of virulence genes *ply* (coding for pneumolysin), *lyt*A (coding for autolysin), *psa*A (coding for pneumococcal surface antigen A)^32–34^, and those coding for pilus type 1 (PI-1)^21^ was investigated by PCR using previously described primers and an automated Veriti 96-well thermal cycler (Applied Biosystems Inc, Carlsbad, CA, USA).

### Evaluation of genetic diversity

Genetic diversity was assessed by Pulsed-Field Gel Electrophoresis (PFGE)^9^, Multiple Locus VNTR Analysis (MLVA)^35^ and Multilocus Sequence Typing (MLST)^36–38^ with modified primers^39^, according to earlier recommendations.

### Phylogenetic analysis

Phylogenetic analysis was performed by Multilocus Sequence Analysis (MLSA)^40^. Concatenates of six *housekeeping* genes (*aro*E, *gdh*, *gki*, *rec*P, *spi*, *xpt*) were generated with an online tool^41^ and a phylogenetic tree was built using Mega X software^42^, using the following parameters: Neighbor Joining algorithm, with bootstrap of 1000, Kimura 2p model and uniform rates of substitutions. There were also included in the analysis nucleotide sequences of typical *S. pneumoniae* strains belonging to STs 1, 2, 3, 4, 5, 7 and 9 (obtained from PubMLST)^37^ and sequences of other *Streptococcus* species (retrieved from GenBank database) including *S. mitis* strain S691 (accession numbers EU075887.1, EU075743.1, EU075784.1, EU075814.1, EU075851.1 and EU075674.1 for genes *aro*E, *gdh*, *gki*, *rec*P, *spi* and *xpt* respectively), *S. pseudopneumoniae* strain 9230 (accession numbers KC491132.1, KC491108.1, KC491058.1, KC491034.1, KC490998.1 and KC490972.1 for genes *aro*E, *gdh*, *gki*, *rec*P, *spi* and *xpt* respectively) and *Streptococcus oralis* strain S527 (accession numbers EU076013.1, EU076236.1, EU075980.1, EU076137.1, EU076186.1 and EU075945.1 for genes *aro*E, *gdh*, *gki*, *rec*P, *spi* and *xpt* respectively).

### Nucleotide sequence accession numbers

The *atp*A gene sequences of the five mutated isolates reported in the present study were deposited in GenBank database under accession numbers KR012497 to KR012501.
